# Potassium management and heart failure: a nephrologist's perspective

**DOI:** 10.1093/ckj/sfae424

**Published:** 2024-12-19

**Authors:** Denis Fouque, Carmine Zoccali, Francesco Pesce

**Affiliations:** Dept of nephrology, Lyon Sud hospital, Hospices Civils de Lyon, Carmen Inserm U1060 and University Claude Bernard Lyon1, Pierre Benite, France; Renal Research Institute, NY, USA; BIOGEM, Ariano Irpino, Italy; Associazione Ipertensione Nefrologia Trapianto Renal (IPNET), C/O Nefrologia, Grande Ospedale Metropolitano, Reggio Calabria, Italy; Division of Renal Medicine, Ospedale Isola Tiberina - Gemelli Isola, Rome, Italy

Potassium serum concentration is tightly and promptly regulated to allow adequate cell functioning, especially for cardiac rhythm control. Beside absorption/release linked to digestive fluid metabolism, potassium behavior depends on multiple regulatory mechanisms, involving the kidney, the skin and the intra-/extracellular exchanges. Various hormones impact on these regulatory loops, among them aldosterone, corticosteroids, catecholamines, insulin, and IGF-1. Likewise, several drugs also alter serum potassium levels, including diuretics, renin–angiotensin–aldosterone system (RAAS) inhibitors, steroidal and non-steroidal mineraloreceptor antagonists (MRA), sodium-glucose co-transporter 2 (SGLT2) inhibitors, potassium binders and substances that alter the acid–base balance, such as sodium bicarbonate, sodium citrate, etc. Dietary intake of potassium comes from different sources and is variable, generally ranging from 1 to 4 g per day. It is increased in diets that are rich in fruits and vegetables, such as the PLADO (plant-dominant low protein) and the Mediterranean diets and generally reduced in western-type diets. A high intake of potassium is associated with a reduction in systemic blood pressure. Interestingly, there has been numerous recent research data showing a much weaker (if any) relationship between dietary potassium intake and kalemia than previously thought. This counterintuitive observation is mostly explained by the difference in potassium intake through either a single chemical compound administered during experimental research (K chloride or K phosphate) which is readily absorbed and quickly enters the bloodstream, and dietary potassium which comes with fibers (legumes), carbohydrates (fruit) and low PRAL (potential renal acid load) brought by most vegetarian diets, all combinations that can neutralize the rise in serum potassium [1]. However, in people with advanced chronic kidney disease (CKD) a relationship may still exist between seasonal substantial potassium intake and kalemia, or obvious dietary error, such as excessive potato, meat or milk portions and potassium additives, which should be assessed by the dietitian. Otherwise, until today there is no proven relationship that reducing dietary potassium can therapeutically improve hyperkalemia [2]. Indeed, in a recent review, a healthy diet including fruits, vegetables and wholegrains was associated with a lower serum potassium [2].

Recently, a debate took place regarding the optimal kalemia and risks of hyperkalemia in patients with cardiac disease taking medications that alter potassium metabolism. Indeed, up to 73% of heart failure patients with CKD may present with hyperkalemia during the first year of follow-up [3–6]. Of note, the level of kalemia and the treatment adjustments differ according to guidelines and publications [7]. New potassium binders have been shown to prevent the recurrence of hyperkalemic episodes under spironolactone treatment, but it is unclear if these binders allow continuation of MRA and improve outcomes [8]. There is a gap in evidence whether these binders should be given preventively in heart failure patients with normokalaemia (<5 mmol/l) as they may induce hypokalemia [8].

Indeed, hypokalemia is also frequently observed in heart failure patients, up to 50% [9], and has been reported to be associated with increased mortality even for mild hypokalemia just below 4 mmol/l [10]. The authors recommend maintaining serum potassium between 4 and 5 mmol/l. Thus, a long-standing relevant clinical question is: what are the goals in serum potassium management, and when and how should we control serum potassium?

**Figure 1:**
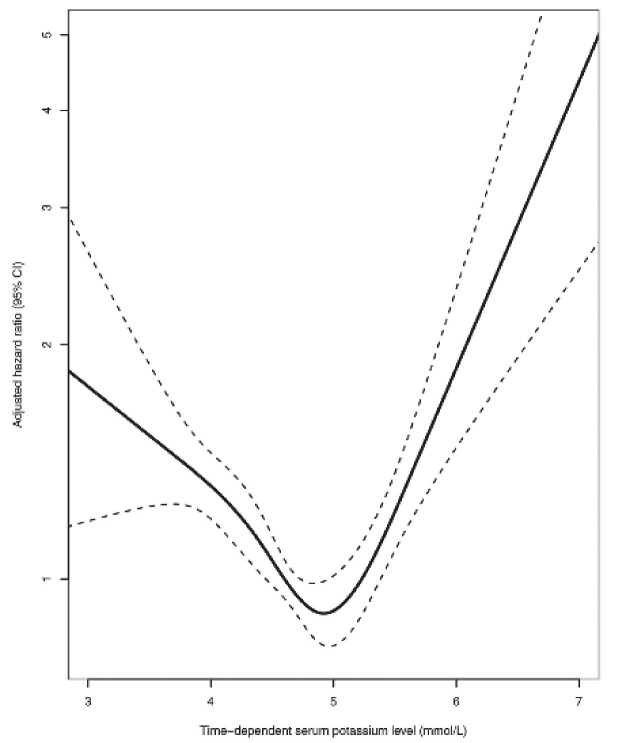
Combined death or kidney replacement therapy start (dotted lines, 95% CIs) related to time-dependent serum potassium in 1714 incident participants in the European Quality study on treatment of older people with advanced CKD during 8 years of follow-up (adjusted for age, sex, current smoking, history of diabetes mellitus, history of cardiovascular disease, estimated glomerular filtration rate, subjective global assessment score, and renin–angiotensin–aldosterone system inhibitor use). From De Rooij et al., ref. 13, with permission.

The serum potassium target in heart failure and CKD patients should be 3.5–5.5 mmol/l (Table 1) and corrective maneuvers are not commended within this range. More frequent serum potassium monitoring can be advised when serum potassium ranges between 4.9 and 5.5 mmol/l [11]. Indeed, we learned long ago from electrophysiology that plasma potassium associated with a normal EKG signal is close to 5.0 mmol/l [12]. Above and below this value, there are slight but abnormal EKG modifications that will be aggravated for values outrunning this threshold. Interestingly, 40 years later, data from the European Quality (EQUAL) study in 1700 CKD stage 4–5 non-dialysis patients concur to the same observation, that a value of 4.9 mmol/l was associated with the lowest mortality [13] (Fig. 1). It is also important to remember that in the presence of elevated potassium, there are numerous reasons for factitious or pseudohyperkalemia, among them a difficult venous puncture, a time lag before actual measurement, hot climate temperature when blood is drawn home and then transported to the laboratory, and test tube hemolysis. In addition, there is a noticeable difference between plasma and serum potassium concentration, serum potassium being greater than plasma potassium by a magnitude of 0.35 mmol/l [14], an important information which has been underlined in the recent KDIGO guideline recommendation 3-11-1-1 [11]. Also, the normal range for serum potassium measurement in biology laboratories/facilities reports is between 3.5 and 5.5 mmol/l. Consequently, one should not take a decision for starting/changing treatment based only on a single potassium measurement. The several confounding parameters should therefore be considered and a double check is often warranted. In our experience, pseudohyperkalemia occurs in 20–30% of elevated kalemia and appears to be normal after a second test. When true hyperkalemia is present, a therapeutic intervention is due, based on the above physiopathology (Table 1) [11].

**Table 1:** Serum potassium range and corrective maneuvers*†.

<3.5 mmol/l: reduce thiazides and loop diuretics, give orange juice and bananas, give potassium supplements
3.5–4.9 mmol/l: maintain treatments
4.9–5.5 mmol/l: maintain treatments, measure serum K more frequently
>5.5 mmol/l: reduce/stop MRA-ACEi-ARA2, increase thiazides and loop diuretics

* Verify laboratory normal potassium range, blood drawing with no tourniquet, no hemolysis present and measure K rapidly after draw; †adapted from KDIGO guideline [11].

Moreover, a number of conditions may induce hyperkalemia and should be corrected in the first place (Table 2) [15]. For instance, abnormal glycemia resulting from insulin deficit or insulin resistance during a septic episode will increase serum potassium; also, acidotic episodes with a serum bicarbonate below 23 mmol/l, as in the case of diabetic patients or in those with advanced CKD [16]. More recently, constipation, which allows an unremitting colic potassium reabsorption, has been shown to be associated with altered potassium metabolism. Consequently, patients using chronic laxatives are less prone to develop hyperkalemic episodes [17].

**Table 2:** The sequential choice in the lowering serum potassium armentarium [15].

1. Increasing urinary potassium excretion (diuretics—SGLT2i)
2. Fighting constipation (dietary fibers—laxatives)
3. Controlling acidosis better (bicarbonate supplement)
4. Binding digestive potassium (oral potassium binders)/correcting dietary potassium excess

An additional key question would be: is there a particular potassium profile in patients affected by heart failure (HF) with reduced ejection fraction (HFrEF)? Indeed, the current therapeutic tools for these patients comprise angiotensin converting enzyme (ACE) inhibitors/angiotensin receptor-neprilysin inhibitors (ARNI), beta-blockers, mineralocorticoid receptor antagonists (MRA) and SGLT2 inhibitors, most of which can alter potassium metabolism; likewise, the use of RAAS inhibitors (RAASi) is recommended in HF with mildly reduced ejection fraction (EF; HFmrEF). Notably, a major issue concerning the use of RAASi is the occurrence of hyperkalemia [18], which complicates the choice of an optimal treatment, particularly in patients with an impaired renal function [19]. Thus, in clinical practice we often find RAASi doses below the maximum target dose in patients with HF [20, 21] and CKD [22–24]. On top of that, the use of diuretics, often hampered by resistance, influences the outcome of such patients. An analysis of the ESC-HFA-EORP Heart Failure Long-Term Registry [25] performed on HF outpatients (60.6% with HFrEF), showed that, when adjusting for RAASi discontinuation, hyperkalemia was no longer associated with mortality, suggesting that the adverse effects of hyperkalemia might be explained by RAASi discontinuation, and that hyperkalemia might be considered as a risk marker for a suboptimal HF treatment.

Potassium binders might give an edge in patients affected by HF with hyperkalemia allowing to maintain a greater proportion of patients on RAASi therapy [18], but this occurrence has been challenged by the DIAMOND trial [26, 27]. The recently published REALIZE-K study aimed at evaluating sodium zirconium cyclosilicate (SZC) to optimize MRA therapy (spironolactone) over 6 months in adult patients with HFrEF and hyperkalemia [28]. The study showed that at 6 months, 71% of patients who received SZC achieved the primary endpoint of normokalemia on spironolactone ≥25 mg/daily without rescue therapy for hyperkalemia compared with 36% in the placebo group (*P* < .001). However, even though overall there were no imbalance in adverse events or serious adverse events, the composite of cardiovascular death or worsening HF occurred more frequently in the SZC (11%) versus the placebo (3%) group. The authors discuss that a few differences in baseline characteristics, such as more advanced heart failure in the SZC group, older age, lower estimated glomerular filtration rate (eGFR), more loop diuretic use, and higher N terminal pro brain natriuretic peptide (NTproBNP) levels, may have potentially contributed to this observation, but nonetheless they conclude that this should be factored into the clinical decision-making.

The treatment with finerenone has proven effective in modifying the CKD-associated composite cardiovascular risk in patients with type 2 diabetes [29]. Finerenone can raise potassium levels as CKD progresses, so monitoring is important. If this happens, the use of diuretics and SGLT-2 inhibitors might be a safe strategy to reduce the risk of hyperkalemia [30]. As for GLP-1r agonists, they typically do not affect potassium levels significantly, although it has been suggested that the risk of heart failure hospitalization may increase in patients with left ventricular ejection fraction (LVEF) < 40% [31]. Therefore, the balance and choice of diuretics remains pivotal, particularly when taking into account the presence or absence of congestion. In fact, both the type of diuretics and the adequacy of dosing are equally important to strike a perfect balance for the heart and the kidney, keeping potassium levels safe while tackling the congestion without the renal function deteriorating.

Finally, how should we manage medications based on serum potassium? The recent KDIGO guidelines in patients with CKD [11] have suggested various interventions to correct serum potassium to its optimal value. The threshold to reduce/stop MRA-ACEi-ARA2 is 5.5 mmol/l, and below this value, no treatment change is required (Table 1). Regular potassium measurements may appear cumbersome; however, they will allow maintenance of cardioprotective medications without adding unnecessary dietary restrictions that may alter patients’ quality of life. Potassium binders, which includes drugs used for decades (sodium polystyrene sulfonate) and others more recently marketed (sodium zirconate and patiromer), are efficient in case of confirmed hyperkalemia episodes (Tables 1–2); however, there is no evidence that a preventive administration may benefit the patients.

It can be argued that with regards to all such clinical questions, what seems to be key is a strict and timed interaction between cardiologists and nephrologists to promptly adjust the relative balance between the cardiac symptoms and the underpinning decline of renal function.
